# Selective Expression of Myosin IC Isoform A in Mouse and Human Cell Lines and Mouse Prostate Cancer Tissues

**DOI:** 10.1371/journal.pone.0108609

**Published:** 2014-09-26

**Authors:** Ivanna Ihnatovych, Neil L. Sielski, Wilma A. Hofmann

**Affiliations:** Department of Physiology and Biophysics, University at Buffalo-State University of New York, Buffalo, New York, United States of America; Northern Institute for Cancer Research, United Kingdom

## Abstract

Myosin IC is a single headed member of the myosin superfamily. We recently identified a novel isoform and showed that the *MYOIC* gene in mammalian cells encodes three isoforms (isoforms A, B, and C). Furthermore, we demonstrated that myosin IC isoform A but not isoform B exhibits a tissue specific expression pattern. In this study, we extended our analysis of myosin IC isoform expression patterns by analyzing the protein and mRNA expression in various mammalian cell lines and in various prostate specimens and tumor tissues from the transgenic mouse prostate (TRAMP) model by immunoblotting, qRT-PCR, and by indirect immunohistochemical staining of paraffin embedded prostate specimen. Analysis of a panel of mammalian cell lines showed an increased mRNA and protein expression of specifically myosin IC isoform A in a panel of human and mouse prostate cancer cell lines but not in non-cancer prostate or other (non-prostate-) cancer cell lines. Furthermore, we demonstrate that myosin IC isoform A expression is significantly increased in TRAMP mouse prostate samples with prostatic intraepithelial neoplasia (PIN) lesions and in distant site metastases in lung and liver when compared to matched normal tissues. Our observations demonstrate specific changes in the expression of myosin IC isoform A that are concurrent with the occurrence of prostate cancer in the TRAMP mouse prostate cancer model that closely mimics clinical prostate cancer. These data suggest that elevated levels of myosin IC isoform A may be a potential marker for the detection of prostate cancer.

## Introduction

Prostate cancer is the most common cancer diagnosed in men worldwide and the second most common cause of cancer death in the US [1], [2]. Prostate cancer can be frequently indolent but it can also be highly aggressive and lethal in some patients. Currently there is no reliable marker available for early detection, diagnostic confirmation, or disease prognosis available [3]. Thus, the identification of novel biomarkers that have the ability to accurately identify and discriminate prostate cancer is of importance for accurate management of the disease.

Myosin IC is a member of the myosin superfamily [4] that localizes to the cytoplasm and the nucleus and is implicated in a variety of processes in both compartments. In the cytoplasm, myosin IC is involved in the formation of lipid rafts [5], transport of vesicles containing membrane proteins [6], and in ion channel regulation in hair cells of the inner ear [7]–[9]. In the nucleus, myosin IC is involved in various aspects of transcription [10]–[13], in chromatin remodeling [14], [15], and in dynamic organization of chromosomal structures [16]. We recently identified a previously unknown isoform of myosin IC and showed that the *MYOIC* gene in mammalian cells encodes three isoforms that are called myosin IC isoforms A, B, and C [17].

Our previous analysis of myosin IC isoform expression in murine tissues revealed a tissue-specific expression pattern of specifically myosin IC isoform A with high expression levels in kidney, adrenal gland, pancreas, and in epididymal and retroperitoneal adipose depots [18]. In contrast, myosin IC isoform B shows a ubiquitous expression pattern in all analyzed tissues and cell lines [17]–[19].

This tissue specific expression of myosin IC isoform A led us to explore the expression profiles of the myosin IC isoforms further. We report here the evaluation of myosin IC isoforms A and B expression in a variety of mammalian tissue culture cell lines. We demonstrate that expression of myosin IC isoform A is significantly increased in prostate cancer cell lines when compared to other (non-prostate) cancer cell lines or to a non-cancer prostate cell line. Subsequent analysis of prostate cancer tumor tissues from the murine TRAMP model that closely mirrors the pathogenesis of human prostate cancer [20]–[23] reveals a significant increase in isoform A expression when compared to normal tissues. Taken together, our data implicate specific changes in myosin IC isoform A expression that are concurrent with the appearance of prostate cancer.

## Materials and Methods

### Antibodies

**Figure 1:**
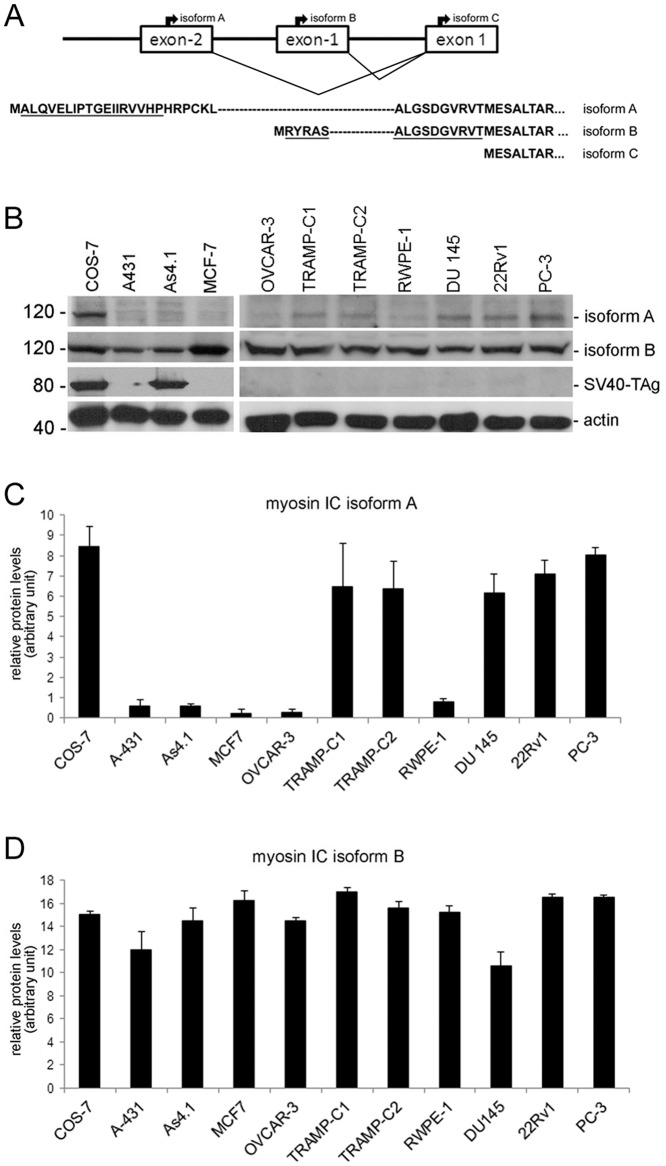
Protein expression of myosin IC isoforms A and B in mammalian cell lines. Detection of myosin IC isoforms A and B protein in various mammalian cell lines by immunoblot analysis using antibodies specific to myosin IC isoforms A, isoform B, SV40 large T-antigen (TAg) and actin (control). **A)** Schematic of myosin IC isoform specific sequences and recognition site of antibodies. Depicted is the 5′ region of the mammalian *MYOIC* gene with the exons that code for isoform specific N-terminal peptides. The translation start sites for the isoforms are indicated as are the N-terminal amino acid sequences. Underlined are the peptide sequences used as immunogen to create isoform-specific antibodies. **B)** Representative immunoblot of cell extracts that were analyzed using the indicated antibodies. Relevant molecular weight markers are indicated on the left in kD. **C)** Histogram presenting the average densitometric intensity of myosin IC isoform A expression normalized to actin. **D)** Histogram presenting the average densitometric intensity of isoform B expression normalized to actin. Myosin IC isoform A protein is highly expressed in COS-7 cells and the human and mouse prostate cancer cell lines PC-3 and TRAMP-C2. In contrast, myosin IC isoform B protein is expressed at comparable levels in all analyzed cell lines. Results are presented as means ± standard deviation; n = 3 independent experiments.

Antibodies that recognize specific isoforms of myosin IC: 1. the anti-NMI antibody is a rabbit polyclonal antibody that was raised against the 16 amino acid long N-terminal peptide of NMI, here called isoform B [5], [24] (Sigma-Aldrich, St Louis, MO); 2. the myosin IC-isoform A antibody is a mouse monoclonal antibody that was raised against the myosin IC isoform A specific N-terminal peptide and recognizes exclusively myosin IC isoform A [17] (Figure 1A). Monoclonal antibodies to β-actin and the SV40 large T antigen (SV40-TAg) were obtained from Sigma (Sigma-Aldrich, St Louis, MO) and EMD Millipore (EMD Millipore Corporation, Billerica, MA), respectively. Peroxidase-conjugated secondary anti–mouse or anti–rabbit antibodies were obtained from Jackson ImmunoResearch Laboratories (West Grove, PA).

### Cell lines and tissue culture

COS-7, A-431, MCF-7, TRAMP-C1, DU 145, 22Rv1, RWPE-1, and PC-3 cells were obtained from American Type Culture Collection (ATCC; Manassas, VA, USA). OVCAR-3 cells, originally obtained from ATCC, were generously provided by Dr. Arthur M. Edelman (Dept. of Pharmacology and Toxicology, University at Buffalo, Buffalo, NY, USA). As4.1 cells [25] were a kind gift from Dr. Kenneth W. Gross [Dept. of Molecular and Cellular Biology, Roswell Park Cancer Institute (RPCI), Buffalo, NY, USA]. TRAMP-C2 cells [26] were a kind from Dr. Barbara Foster (Dept. of Pharmacology and Therapeutics, RPCI, Buffalo, NY, USA). COS-7, A431, and As4.1. cells were grown in Dulbecco’s modified Eagle’s medium (DMEM) supplemented with 10% fetal bovine serum (FBS). MCF-7 and DU 145 cells were grown in DMEM supplemented with 10% FBS and 0.01 mg/ml insulin (Sigma-Aldrich, St Louis, MO). PC-3 and 22Rv1 cells were grown in RPMI media supplemented with 10% FBS. OVCAR-3 cells were grown in RPMI media supplemented with 20% FBS and 0.01 mg/ml insulin. TRAMP-C1 and TRAMP-C2 cells were grown in DMEM high glucose supplemented with 5% FBS, 5% Nu Serum IV (BD Biosciences, San Jose, CA, USA) 5 mg/mL insulin and 10 nmol/L Dihydrotestosterone (Sigma-Aldrich, St Louis, MO, USA). RWPE-1 cells were grown in Keratinocyte Serum Free Medium (Invitrogen Carlsbad, CA, USA; Kit Catalog # 17005-042) supplemented with 0.05 mg/ml bovine pituitary extract (BPE) and 5 ng/ml human recombinant epidermal growth factor (EGF). In addition, all cells were supplemented with 1% penicillin/streptomycin and grown at 37°C with 5% CO_2_.

### Tissue samples

Frozen tissue samples from 22 weeks of age TRAMP mice as well as age matched wild type mice were purchased from the Mouse Tumor Model Resource at RPCI (Buffalo, NY, USA). The tissues were collected in strict accordance with the recommendations in the Guide for the Care and Use of Laboratory Animals of the National Institutes of Health under RPCI’s 890M Tumor banking protocol for the Mouse Tumor Models Resource. The protocol was approved by Roswell Park Cancer Institute’s IACUC (Institutional Animal Care and Use Committee). Animals were sacrificed by anesthesia overdose followed by cervical dislocation. All prostate and tumor tissues had been microdissected at necropsy, flash-frozen in liquid nitrogen, and stored at −80°C until use. Five-micron-thick paraffin embedded matched tissue sections were purchased from the same source.

### Immunohistrochemistry

Paraffin embedded tissue sections were de-paraffinized with xylene, rehydrated with alcohol, and placed in dH_2_O before undergoing standard antigen retrieval (citrate buffer, pH 6). Endogenous peroxidase activity was blocked by using 3% H_2_O_2_ prior to incubation with either myosin IC isoform A or SV40-TAg antibodies for immunohistochemistry according to standard methods. Images were obtained with a Zeiss Axio Imager Z1 (Carl Zeiss Inc, Thornwood, NY, USA) and processed using Adobe Photoshop software.

### Immunoblot analysis

For preparation of crude cellular extract, an equal number of cells was lysed in SDS sample-buffer. For each cell line based analysis, three independent experiments were performed. Tissue extract was prepared as described previously [18]. For each tissue based analysis, tissues from three to six mice were analyzed. Equal volumes of crude cell extract or equal amounts of tissue protein extracts (40 µg) were separated by 10% SDS-PAGE (sodium dodecyl sulfate-polyacrylamide gel electrophoresis) and transferred onto nitrocellulose membrane. After transfer, the membrane was cut at appropriate sizes and incubated with specific antibodies. Immunoreactive bands were detected by enhanced chemiluminescence. Densitometric analysis was performed on the selected bands based on their relative intensities using ImageJ software.

### Quantitative Real-Time PCR (qRT-PCR)

Total RNA was isolated from cells or tissue samples using Trizol reagent (Invitrogen, Carlsbad, CA, USA) following the manufacturer’s instructions. 1 µg total RNA from each cell line was used to reverse transcribe into cDNA using Superscript III reverse transcriptase and oligo dT primer according to manufacturer’s instructions (Invitrogen, Carlsbad, CA). qRT-PCR was performed using the iCycler iQ Real-Time PCR Detection System (Bio-Rad, Hercules, CA, USA) and primer that recognize specifically mouse and human myosin IC isoform A (*homo sapiens:* NM_033375.4; *mus musculus*: XM_006532429.1) and mouse and human myosin IC isoform B (*homo sapiens:* NM_001080950.1; *mus musculus:* NM_001080775). For quantification, triplicates were normalized to the average of the GAPDH housekeeping gene. The primer used for qRT-PCR are described in [18].

### Statistical analysis

All data are given as means ± standard deviations. The normality of the data was tested with the Kolmogorov-Smirnov test. The difference between data sets was tested with Student’s t-test. The deviations were considered significant at the level of *p* < 0.01.

## Results

We previously analyzed the expression patterns of myosin IC isoforms in 33 mouse organs and tissues and found that myosin IC isoform A but not isoform B is expressed in a tissue specific manner [18]. We now extended our analysis of the protein and mRNA expression of myosin IC isoforms to a panel of mammalian cell lines. To analyze protein expression, we performed immunoblot analysis of total cell extracts using myosin IC isoform–specific antibodies (Figure 1A). As shown in Figure 1B and C, besides in the African green monkey kidney cell line COS-7 (where it was originally detected and characterized [18]) myosin IC isoform A protein is highly expressed in the human and mouse prostate cancer cell lines TRAMP-C1, TRAMP-C2, DU 145, 22Rv1, and PC-3, but not in the non-cancer prostate cell line RWPE-1. Furthermore, myosin IC isoform A shows only low expression levels in other cancer cell lines including human skin (A-431), breast (MCF-7), and ovarian (OVCAR-3) cancer cell lines. Because the COS-7 cell line was derived by transformation with an origin defective mutant of SV40 that still encodes the SV40 large T antigen (SV40-TAg) [27], we also tested the As4.1 cell line that was established from cells of a transgenic mouse with an intraparenchymal kidney tumor that was induced by targeted tumorigenesis with an SV40-TAg fusion construct and also expresses SV40-TAg [25]. As shown in Figure 1A, both cell lines, COS-7 and As4.1, express SV40-TAg but only COS-7 cells show a high expression level of myosin IC isoform A. In contrast to this cell line-specific expression of myosin IC isoform A, myosin IC isoform B is ubiquitously expressed at comparable levels in all analyzed cell lines (Figure 1A&B).

**Figure 2:**
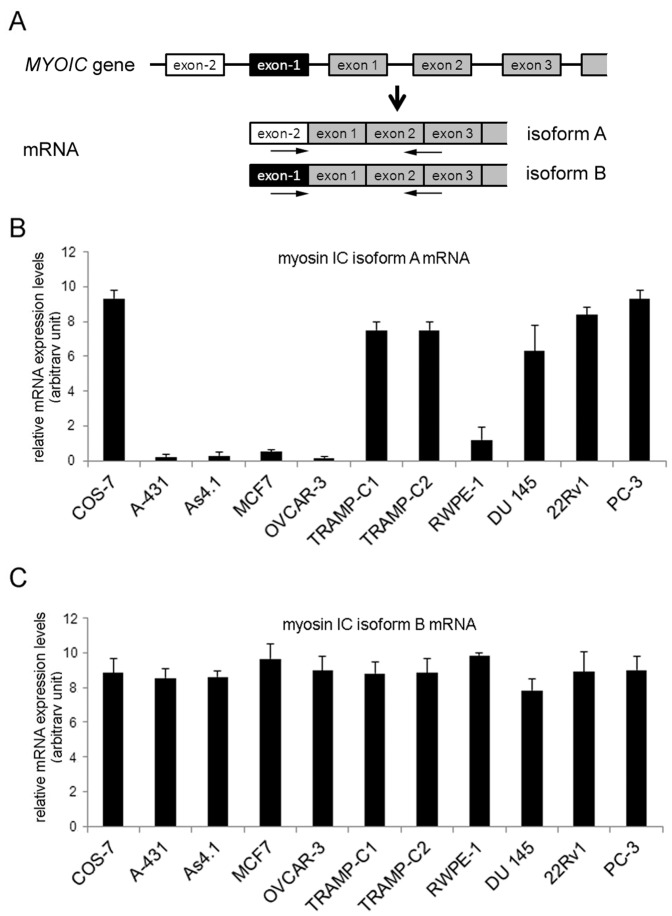
mRNA expression of myosin IC isoforms A and B in mammalian cell lines. Detection of myosin IC isoforms A and B mRNA levels in various mammalian cell lines by qRT-PCR using isoform-specific primer. **A)** Schematic depicting the 5′ region of the *MYOIC* gene and the resulting isoform A and B mRNAs. The target sequence location of primer used for qRT-PCR to detect myosin IC isoforms are indicated by arrows. **B)** Quantitative real-time PCR analysis of mRNAs expression levels of myosin IC isoform A normalized to GAPDH. **C)** Quantitative real-time PCR analysis of mRNAs expression levels of myosin IC isoform B normalized to GAPDH. Myosin IC isoform A mRNA expression is high in COS-7 cells and the human and mouse prostate cancer cell lines PC-3 and TRAMP-C2. In contrast, myosin IC isoform B mRNA is expressed at comparable levels in all analyzed ell lines. Results are presented as means *±* standard deviation; n = 3 independent experiments.

To determine if protein expression correlates with mRNA expression, we next analyzed mRNA expression of the isoforms using quantitative real time PCR (qRT-PCR) with isoform-specific primer (Figure 2A). As shown in Figure 2, we found that the mRNA expression profiles of myosin IC isoforms A and B correlate to the protein expression profiles. Comparable to the protein data, mRNA expression of myosin IC isoform A is only high in COS-7, TRAMP-C1, TRAMP-C2, DU 145, 22Rv1, and PC-3 cells while myosin IC isoform B mRNA is ubiquitously expressed in all analyzed cell lines.

A striking observation of our analysis of myosin IC isoform expression in these cell lines are the high expression levels of myosin IC isoform A in the mouse and human prostate cancer cell lines when compared to the normal (non-cancer) prostate cell line RWPE-1 and to other (non-prostate) cancer cell lines as summarized in Table 1. This is particularly interesting because our previous analysis of mouse tissue and organs showed a very low expression of myosin IC isoform A in normal mouse prostate [18]. In combination with the the low expression levels of isoform A in the human normal prostate cell line RWPE-1, these data suggest that changes in myosin IC isoform A expression could be associated with the development of prostate cancer.

**Table 1:** Myosin IC isoform A expression in mammalian cell lines.

Cell line	Description (tissue, disease)	Species	myosin IC isoform Ahigh expression levels
COS-7	kidney; SV40 transformed	*cercopithecus aethiops*	**yes**
As4.1	kidney/ascides fluid of transgenic mouse with kidney tumor	*mus musculus* (transgenic)	no
TRAMP-C1	prostate; adenocarcinoma	*mus musculus* (transgenic)	**yes**
TRAMP-C2	prostate; adenocarcinoma	*mus musculus* (transgenic)	**yes**
A-431	skin/epidermis; epidermoid carcinoma	*homo sapiens*	no
MCF-7	mammari gland, breast; derived from pleuralmetastatic site; adenocarcinoma	*homo sapiens*	no
OVCAR-3	ovary; adenocarcinoma	*homo sapiens*	no
RWPE-1	prostate; normal	*homo sapiens*	no
PC-3	prostate, derived from bone metastatic site; adenocarcinoma grade IV	*homo sapiens*	**yes**
DU 145	prostate; derived from brain metastatic site; carcinoma	*homo sapiens*	**yes**
22Rv1	prostate; carcinoma	*homo sapiens*	**yes**

To explore this possibility, we analyzed myosin IC isoform A expression in various tissues from the TRAMP mouse. TRAMP is a transgenic line of C57BL/6 mice that express a construct containing the minimal −426/+28 regulatory element of the rat probasin promoter to target expression of the SV40 large T antigen to prostatic epithelium [21]. The TRAMP model is a widely used prostate cancer model as it mimics closely human prostate cancer. Spontaneous tumor progression in the TRAMP prostate begins at 8 to 12 weeks of age, and 100% of mice develop prostate cancer by the age of 20 weeks. Subsequently, metastasis can occur at distant sites as the tumors progress [20], [28], [29].

**Figure 3:**
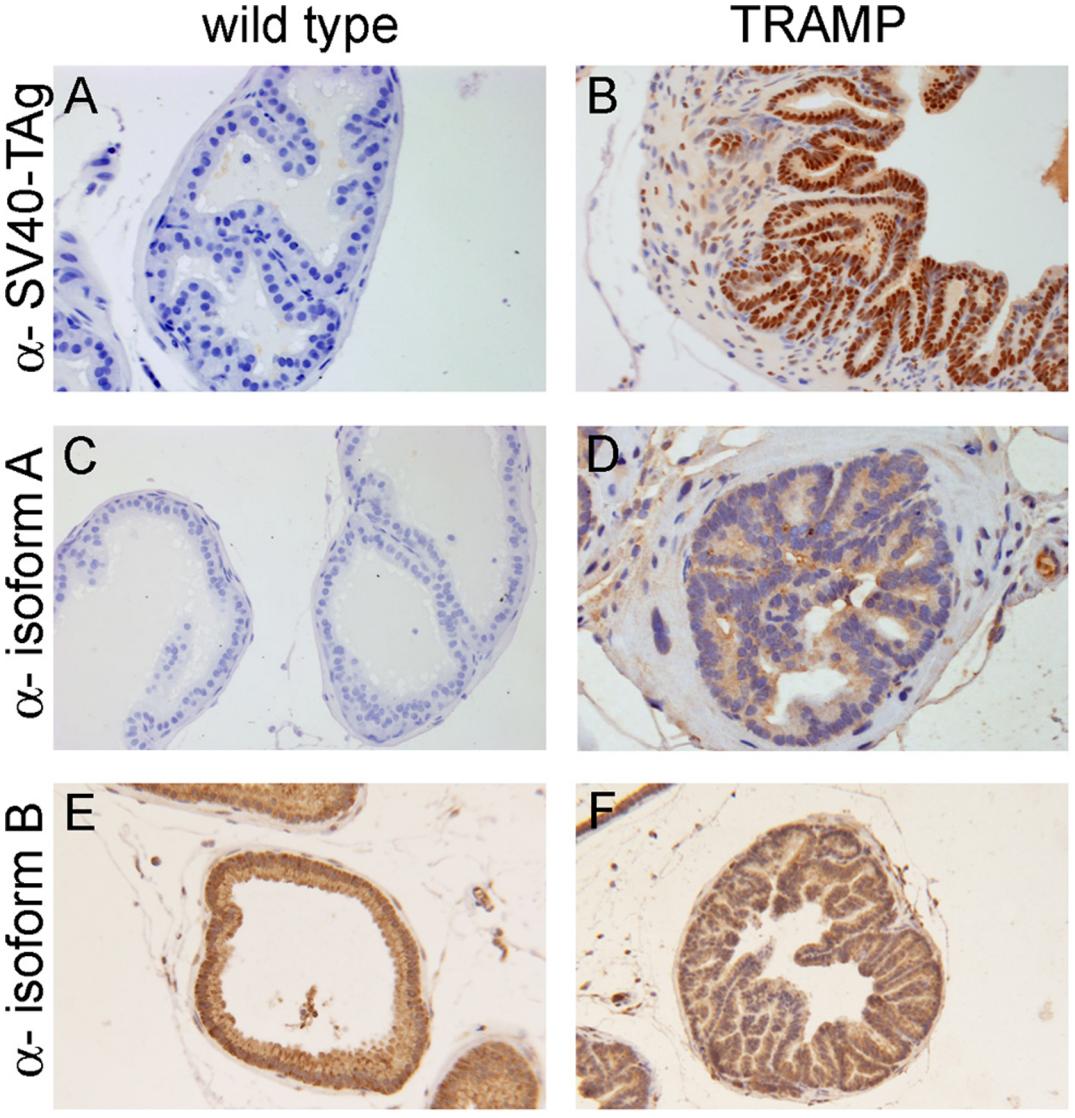
Expression of myosin IC isoform A in prostate tissue. Representative image of immunohistochemical analysis of SV40-TAg (A&B), myosin IC isoform A (C&D), and isoform B (E&F) expression in prostate glands of 22 weeks of age wild-type (left column) and TRAMP (right column) mice. The slides were counterstained with hematoxylin. Images were taken at 20x magnification. The histological changes of the TRAMP mouse prostate that are associated with PIN lesions are obvious (left column, B, D, F). As expected, SV40-TAg immunostaining is negative in wild-type prostate (A) and strongly nuclear in TRAMP prostate (B). Myosin IC isoform A shows very weak immunostaining in wild type prostate tissue (C) but strong, predominantly cytoplasmic staining in TRAMP prostate with PIN lesions (D). Myosin IC isoform B shows strong staining in both, wild type and TRAMP, prostate tissue (E&F).

Figure 3 shows the immunohistochemical analysis of representative wild type and TRAMP mouse prostate from mice of 22 weeks of age. The TRAMP tissues exhibit typical morphological features that are associated with PIN lesions and stain positive for nuclear SV40-TAg [23] (Figure 3A&B). Our analysis of myosin IC isoform expression in these tissues revealed a markedly increased presence of myosin IC isoform A in cells associated with characteristic PIN lesions of TRAMP mice when compared to cells of wild type prostate tissue in which myosin IC isoform A is barely detectable (Figure 3C&D). In contrast, both normal and TRAMP prostate tissue stain positive for isoform B (Figure 3 E&F).

**Figure 4:**
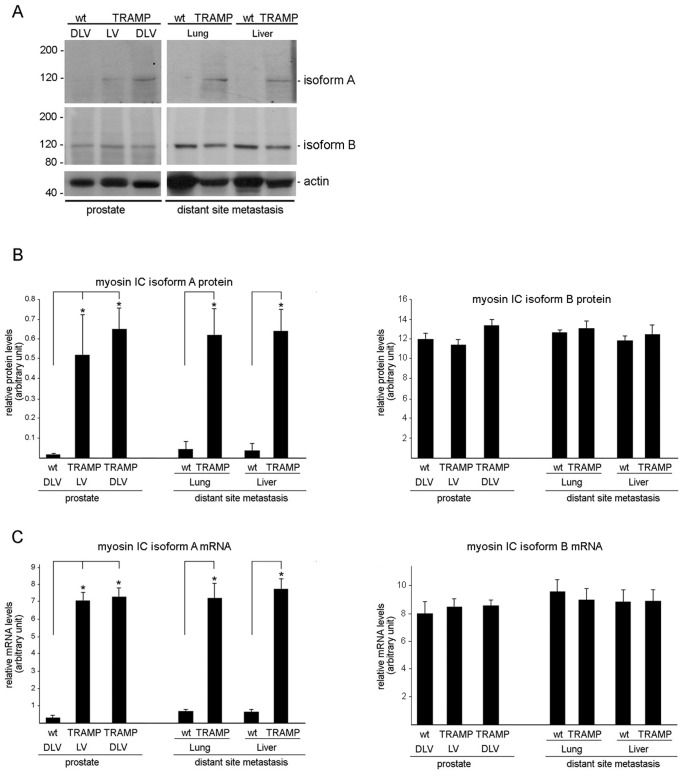
Protein and mRNA expression of myosin IC isoform A in prostate and tumor tissues of wild type and TRAMP mice. Prostate tissue extracts consisting of dorsal, lateral, and ventral (DLV) or lateral and ventral (LV) prostate from 22 weeks of age TRAMP or age matched wild type mice were analyzed for protein and mRNA expression as were tissue extracts from lung and liver metastases of TRAMP and matched tissues of wild type mice. A) Representative immunoblots of mouse tissue extracts that were analyzed using the indicated antibodies. Relevant molecular weight markers are indicated on the left in kD. B) Histogram presenting the average densitometric intensity of isoform A expression normalized to actin. C) Quantitative real-time PCR analysis of mRNAs expression levels of myosin IC isoform A normalized to GAPDH. Results are presented as means ± standard deviation; n = 3–6 (number of tissues tested each from a different animal). *p<0.01 compared to normal tissue.

To confirm the presence of myosin IC isoform A in tissues associated with prostate cancer, we analyzed various TRAMP prostate tumor tissues that were comprised either of lateral and ventral (LV) or of dorsal, later, and ventral prostate (DLV). As shown in Figure 4, myosin IC isoform A protein levels were significantly higher in prostate tumor tissues from TRAMP mice when compared to wild-type prostate tissues that show only barely detectable myosin IC isoform A expression levels (Figure 4A&B and [18]). In addition, we analyzed tumor tissues from distant site metastasis, i.e. lung and liver tumors. Similar to the prostate tissues, liver and lung tumors showed a significant increase in myosin IC isoform A protein expression when compared to the expression levels in wild type normal lung and liver tissues [Figure 4A&B and [18]]. Further examination of myosin IC isoform A expression by qRT-PCR confirmed that the increased protein expression in TRAMP prostate and distant site metastases correlates to a significant increase of myosin IC isoform A mRNA expression in these tissues (Figure 4C).

## Discussion

An analysis of myosin IC isoform A expression in prostate tissue and distant site metastases in TRAMP mice or age matched wild type mice showed that myosin IC isoform A is significantly higher expressed in prostate tumors relative to normal murine prostate, lung, or liver tissues. This is, to our knowledge, the first report documenting a change of a myosin IC isoform expression in connection to cancer. The possible biological and physiological consequences of the overexpression of myosin IC isoform A in tumor tissues are currently under investigation in our laboratory.

In addition to establishing a link between myosin IC isoform A expression and cancer, our data suggest that aberrant myosin IC isoform A expression changes can be specifically linked to transcriptional changes associated with the occurrence of prostate cancer. This hypothesis is supported by several observations. Our previous analysis of myosin IC isoform A expression in mouse tissue samples [18] revealed that this specific isoform shows high expression only in kidney, adrenal gland, pancreas, and a subset of adipose tissues. We now show a significantly increased expression in prostate, lung, and liver prostate cancer tumor tissues from the TRAMP mouse when compared to wild-type tissues.

The TRAMP prostate cancer model is based on the prostate-specific expression of the SV40-TAg oncoprotein [21]. The large T antigen of SV40 acts through several pathways including inactivation of tumor suppressor proteins p53 and Rb as well as transcriptional activation of a number of proteins involved in oncogenesis [22]. Because SV40-TAg is a universal rather than a prostate cancer-specific oncogene that has been used extensively as a model of many other cancer types [30], it could have been possible that changes in myosin IC isoform A expression were induced through the actions of SV40-TAg regardless of cancer type. However, our analysis showed that isoform A expression was not altered in the As4.1 cell line that was established from the ascites fluid of a six month old transgenic mouse with an intraparenchymal kidney tumor that was induced by transgene targeted tumorigenesis through SV40-TAg fused to the renin promoter [25]. Furthermore, we found myosin IC isoform A expression altered in the TRAMP-C1 and TRAMP-C2 cell lines that, although originating from TRAMP mice, do not express SV40-TAg [26]. Moreover, we also found increased isoform A expression in the SV40-TAg independent human prostate cancer cell lines PC-3, DU 145, and 22Rv1 but not in the non-cancer human prostate cell line RWPE-1 or in any of the other non-prostate cancer cell lines. Taken together, these data strongly suggest that myosin IC isoform A expression changes are concurrent with prostate cancer specific alteration in cellular expression profiles rather than SV40-TAg-specific expression changes.

While the expression pattern of myosin IA isoform A in human and mouse prostate cancer needs to be analyzed further, our results here, that show an overexpression in human and mouse prostate cancer cells lines and in prostate cancer associated murine tissues, strongly suggest that specifically myosin IC isoform A could be a possible diagnostic marker for detection of prostate cancer.
